# Hospital and Institutional News

**Published:** 1919-02-08

**Authors:** 


### HOSPITAL AND INSTITUTIONAL NEWS.

### THE MINISTRY OF PENSIONS.

Our article, "The Ministry of Pensions: Prompt Reorganisation Essential" in The Hospital of the 4th ultimo has been followed by the announcement that Sir Laming Worthington Evans, the Minister of Pensions just appointed, has decided to make certain changes in the administration of the medical branch of the Ministry. The end in view has been the transfer of hospital accommodation, and medical personnel for disabled men, from the military authorities to the Pension Ministry, for the success of which it is essential to ensure the closest co-operation between the Navy, the Army, the Air Force, and the Ministry. Sir L. Worthington Evans has accordingly appointed Colonel Webb, at present Assistant Director of Medical Services, War Office, to be Director-General. Under him there will be three Directors of Medical Service, each at the head of a definite branch of the work of the Department. Colonel Webb is admittedly one of the ablest officers in the service. Sir John Collie will resume his medical work for the London County Council, and has been invited, and has consented, to act as consulting medical officer to the Ministry on resigning his appointment as Director of Medical Services.

### ENCOURAGING THE STUDY OF CHILDREN'S DISEASES.

Two interesting lectureships have been founded in connection with the Royal Hospital for Sick Children on which the directors may congratulate themselves. The founders are Mr. Leonard Gaw, a Glasgow shipowner, and Mr. Robert Francis Barclay, for twenty years honorary secretary to the hospital. Each has offered a sum of £5,000 to found a lectureship in his name; Mr. Gaw's on "The Medical Diseases of Infancy and Childhood," and Mr. Barclay's on "Surgery and Orthopædics in relation to Infancy and Childhood." The conditions of both endowments are interesting. It is laid down that the lectures shall be both systematic and clinical," that the lecturers shall be respectively a visiting physician and a visiting surgeon to the hospital in the wards of which the clinical instruction shall be given, and that the terms of appointment shall be arranged between the University Court and the directors of the hospital. Further, the medical lecturer is to have "a consultative connection" with the Royal Maternity Hospital "so that children born there and requiring medical or surgical treatment may be brought directly into touch with the hospital in Yorkshire." Children's diseases and orthopædics have both been somewhat neglected in the past, and though the war has done much to intensify the study of the latter, the new lectureships should give a stimulus to the study of children's diseases which are well worth further attention.

### A DELUDED MEDICAL STAFF.

A special meeting of the Governors of Enfield Cottage Hospital was held on 17th ult., to consider the position created by the refusal of the medical staff to sign medical certificates in respect of patients on their admission. This is a direct breach of the rules which the Council, by whom the medical staff is appointed and can be dismissed, is entrusted to administer. The action of the doctors originated in the admission by one of their number, Dr. Ridge, of a patient who was not resident in the district. Her consequent ineligibility was concealed only by the fact that she was the sister-in-law of a householder near by. When the Council courteously asked Dr. Ridge whether there were any special circumstances to justify his action, he did not reply, but, as events proved, endeavoured to screen himself behind his colleagues. For a letter was received from the hon. secretary of the medical staff saying that "the doctors maintained their right to admit cases when necessary irrespective of their residence." Under the rules emergency or accident is the only justification for the admission of non-residents; and the admission of patients rests, not with the medical staff, but with the Council. In fact, the members of the medical staff have been deluded by Mr. Ridge to disobey the rules under which they accepted their appointment, and to defy the Council. As he shrank from attempting to justify his previous conduct, so his colleagues have since declined even to submit alterations in the rules. In short they have allowed themselves to be put by him in the wrong from start to finish. If the Council has erred it has erred on the side of forbearance, for it possesses an undertaking signed by him "to abide strictly by all the rules of the hospital." His action invites dismissal, and his medical colleagues must surely by this time have learnt that they have everything to lose and nothing to gain by remaining in the false position into which he has led them.

### ALLEGATIONS AGAINST THE AIR MINISTRY.

The Lambeth Board of Guardians has referred a dispute with the War Office to the Local Government Board. It appears that Mr. James Kyle, temporary assistant medical officer at the infirmary, was summoned to attend at the Air Ministry, where he was informed that he had been nominated for a commission as a medical officer. He was called upon to sign the necessary papers under threat that if he did not do so his nomination would be cancelled, and he would be conscripted as a private. He was also informed that, because of the present difficulties at the infirmary, he would be allowed to remain there until the end of March. As the papers were not in order, Mr. Kyle was unable to sign them, but the documents were to be sent to him for his signature. He had served two years in the Army as a combatant officer in France before he was released from service to continue his medical studies, being appointed clinical assistant at the infirmary, and qualifying in October last.

### DR BALY DEFENDS HIS STAFF.

The Medical Superintendent, Dr. Lionel Baly, in reporting these facts to the Board, added that he was under the impression that the Local Government Board had complete control of the Poor-Law medical service, and that any demand for medical men to be given up should be made through the Board. He had, therefore, requested Mr. Kyle to leave the contract with the Air Ministry in his hands in order that the guardians might have an opportunity of approaching the Local Government Board on the subject. This the guardians decided to do, with a request that the Board should intervene on their behalf to ensure the service of Mr. Kyle being retained. If the facts bear their face value the Air Ministry has grossly exceeded its province, and threats of the kind mentioned should be visited upon their authors. We hope that a full enquiry will be made.

### THE DEATH OF DR. BELL.

With deep regret we announce the demise of Dr. Edward Scott Bell, M.R.C.S. (Eng.), L.R.C.P. (Lon.), M.D. (Brux.). It appears that Dr. Bell, who was 52 years old, was in the habit of sniffing chloroform to induce sleep, and that he suffered from heart trouble. Death was due to heart failure, and at the inquest a verdict of "Misadventure," was returned. Mr. Bell, during the many years he has been associated with Bermondsey Infirmary, has shown rare skill and knowledge, and a love for the poor. He successfully initiated and carried out a series of reforms in the medical attendance and nursing of the outdoor sick poor. In one of the poorest of London boroughs, Dr. Bell will be greatly missed.

### "ELIMINATING THE V.A.D."

The Ministry of Pensions has many hospital cases on its hands, and it does not seem that its arrangements with the voluntary hospitals will suffice for their treatment. Is the Ministry, therefore, to retain those so far managed by the V.A.D.s? Colonel C. W. Carr-Calthrop, Director of Voluntary Aid Detachments for Middlesex, in informing the Dollis Hill Hospital, Willesden, that the Ministry of Pensions did not wish to retain the institution, has expressed the opinion that the Ministry would rather eliminate the V.A.D.s and start small hospitals of its own, equipped and managed by its own paid staff.

### THE WILLESDEN WAR MEMORIAL.

The proposal to build a General Hospital for Willesden on the freehold site which adjoins the present premises is now well launched. The King's Fund has consented to the issue of an appeal, which, along with other appeals, was deliberately postponed during the war; the sum of £3,500 has been given in for subscriptions and finance, building, and publicity committees have been appointed. It is fifteen years since the land next the cottage hospital was purchased, and the sum of £100,000 is now asked for, of course, as Willesden's memorial to those who have fallen in the war.

### HOSPITAL SALES.

Now that many Red Cross hospitals are closing, a series of sales of beds, bedding, lockers, and equipment of all kinds is taking place throughout the country. It is a pity that a list of these sales cannot be advertised so that other permanent institutions with purchases to make can have the opportunity to benefit by them. No doubt there will be no lack of local purchasers. Dealers, shopkeepers, and provident people who wish to make advantageous purchases of mattresses and beds, of which the trade prices are now high and the supply uncertain, will doubtless take full advantage of these sales. But there must be many hospitals which could also add judiciously to their depleted stocks by purchasing at second hand equipment and bedding whose history is known and which, in many cases, is hardly the worse for wear. As it is, we fear that the opportunity will be lost, and that the only people to benefit will be, generally speaking, local dealers.

### THE NEW BECCLES HOSPITAL.

The people of Beccles, having decided unanimously to build a new hospital for the town, the general committee has appointed the Mayor to be chairman, Mr. J. P. Larkman treasurer, and Mr. W. B. Forward secretary. To unite the district in support of the scheme, Dr. Wood-Hill has drafted a scale which will entitle donors to name beds or wards in the new building.

### SOME EARLY HOSPITAL REPORTS.

Almost the first returns of hospital finances and statistics for 1918 we have received come from the provinces, the Dewsbury General Infirmary beating the Ancoats Hospital by a few days. It is curious that these institutions, which are not obliged to produce figures by a given date, as are the Metropolitan hospitals, should generally be the first to supply their supporters with an account of their stewardship. This early production of figures is always possible if interim balancing of books and the keeping of records written up to date is arranged, and, as auditors are most busy in the first months of the year, the taking of time, in this manner, by the forelock is doubly desirable. It is pleasant to see that the stupendous increases in the cost of maintenance have at last been arrested, and it is hoped that voluntary hospital expenditure will now quickly fall to something like normal figures. The total ordinary expenditure of Ancoats Hospital increased from £14,168 in 1917 to £15,665 in 1918. This rise of £1,500 would ten years ago have been looked upon with great uneasiness, but as during the war the cost of working a hospital of this size often rose by three or four thousand pounds in a single year, this latest increase seems comparatively negligible. Both at Dewsbury and Ancoats, the hospitals have managed to pay their way during 1918, and also appreciably improve the cash balances in hand.

### VOLUNTARY HOSPITALS AND SERVICE MEN.

Once again an inquest has brought to light the rule which ordinarily debars a voluntary hospital from admitting a soldier. When a soldier meets with an accident, seeks admission to a voluntary hospital, is passed on to one, or perhaps two, military hospitals where, as likely as not, he is kept waiting before being properly examined, and then dies, public sympathy is inevitably with the soldier. The latest instance of this has occurred at Exeter. Evidence showed, however, that the unfortunate man could not have lived, and the defence of the Exeter Hospital was, first, the rule, and secondly that in cases where the degree of injury is not easily apparent, the less the patients are handled the better. Temporary treatment, however, is always given where a loss of blood is taking place; in fact, the hospital is encouraged to admit these cases as, in certain circumstances, a liberal grant is given by the War Office for their treatment. If an injured soldier is brought to the hospital at night he is always admitted. This explanation implies a reasonable interpretation of the official rule, but voluntary hospitals should do their best to give an injured service man the benefit of any doubt, since the military hospitals seem to be slack in the admission and treatment of accidents.

### SHOULD WE WEAR FACE MASKS?

Medical men are as brave as most people. Sometimes they are braver. What could be more courageous, for instance, than the suggestion made by Captain Thomas Carnwath, D.S.O., M.B., at the Royal Institute of Public Health, that face masks should be adopted for general use by the public as a protection against influenza? People had learned to use umbrellas, he said, and he did not see why they should not also learn to wear face masks. Umbrellas and face masks are hardly on the same plane, but Captain Carnwath's point is easy to grasp. Masks might do much to prevent the spread of influenza, but unfortunately they are not things of æsthetic joy. Human Nature in the bulk is a vain, frivolous creature, with a yearning for the beautiful. As masks would hide pretty faces, it is to be feared that people would prefer to risk the deadly darts of the influenza microbe. That is foolishness, no doubt; but when did Fashion ever bow the knee to Health? The only prospect we can see of European races going behind the veil, in times of epidemic or at any other period, is for Captain Carnwath and his disciples to enlist the support of the arbiters of Fashion. All will then be plain sailing.

### A PIONEER OF INFANT WELFARE.

One of the great aims of health reformers is to bring down the high rate of infant mortality, which at present in the twenty largest towns stands at 107 per 1,000. Dr. A. Mearns Fraser, medical officer of Health for Portsmouth, who is himself a pioneer in child welfare work, thinks that if local authorities wisely employ the new powers conferred upon them by the Maternity and Child Welfare Act this holocaust of the innocents could be reduced to 50 per 1,000. One of the most important steps, in his opinion, is the provision of municipal lying-in hospitals, to be used as active centres from which would radiate all efforts in connection with the care of motherhood. The establishment of lying-in hospitals in the larger towns would also, he urges, supply a place where women who wish to qualify as midwives could receive their training, instead of having, as now, to go to London or other places where such hospitals are in being. In connection with such lying-in hospitals, Dr. Fraser advocates wards where babies in need of skilled medical treatment could be cared for, and where the offspring of unmarried mothers might find a home. Portsmouth can point with pride to the good results which have followed the serious attention paid to infant welfare in the past. Whereas 20 years ago the infantile mortality in the town was 165 per 1,000, it has been steadily reduced until, in 1918, it stood at the low figure of 74, and in the year before that at only 70. This record carries its own significance; no other large town in the country can compare with Portsmouth.

### CLUB LIFE AND MATERNITY.

A surprising extension of work is shown by the Leeds Maternity Hospital, which the honorary secretary, Mrs. Bartlett, claimed to be twice as extensive as that performed five years ago. The hospital now provides the maternity training for all the medical students of Leeds University; and the nurses and pupils in training last year numbered thirty-four. One speaker at the recent annual meeting, Mrs. A. B. Fisher, declared that the war had encouraged an unprecedented growth in club life, which affected women as well as men, and she feared that motherhood would take a secondary place in many women's desires. Exactly what Mrs. Fisher meant by this danger of club life is not apparent from the report of her remarks which has reached us. Impatience with domestic life is, however, a symptom of the times, and it remains to be seen how the women who have been at work in factories during the war will give up the habits which, according to reliable reports, made even the ex-cotton spinners in the Army long above all things to get back to the Lancashire mills.

### THE POOR-LAW GARDENS.

Not much is heard of gardens in connection with Poor-Law Institutions, but they exist and regard themselves as sources of profit as the following appointment will show. The Garden Committee of the Steyning Board of Guardians has obtained the approval of the board to appoint Mr. Cuddington to superintend the workhouse garden for one year at a salary of £100 together with 5 per cent. commission on all sales up to the value of £500. Should that total be exceeded the commission is to be reconsidered. A foreman gardener is also to be employed as an assistant, and a third, the present working foreman, will superintend the inmates employed on horticulture. It would be interesting to have the accounts of these Poor-law gardens to see to what extent they have become profitable as well as pleasant.

### A TRIBUTE TO INDIAN DOCTORS.

The case for the issue of joint hospital appeals finds more favour, as is natural, in the comparatively self-conscious centres in the Provinces than the amorphous accretion of London. But it has been suggested that the Manchester hospitals should adopt the plan to aid them in the difficulties created or increased by the war. Among these has been the staff difficulty. It is well, therefore, to place on record the tribute which Mr. P. W. Kessley, Chairman of the Manchester Eye Hospital, has paid to Indians. The eye hospital, he remarked lately, was forced to fall back upon fourth and fifth-year medical students from the universities, and to secure the help of a number of foreign students, most of whom were Indians. The present resident surgical officer is an Indian, and Mr. Kessley remarked on the excellent service which had been rendered by the Indian members of the staff.

### BY AIRSHIP TO LONDON.

Mr. Martin, a member of the Stonehouse Board of Guardians, in discussing arrangements for the Board's delegates to attend the Central Poor Law Conference in London, suggested that the delegates should travel to the Metropolis by airship. The Chairman said that he would not shrink from such a journey, and it remains only for the airship to be secured and the cost of the journey to be compared with the old method of railway travelling.

### THE SOLDIERS' CHOICE.

The soldiers whose educational needs are being looked after at the Great Northern Central Hospital sometimes choose a subject on which they wish to hear a lecture. Last week, on January 31, they chose "George Meredith," on whom Professor G. Currie Martin delivered an address. The choice is interesting because that author's reputation seems to have declined since his death. Collected editions of his books are a favourite wedding present, but books for wedding presents, like books for prizes, are not often those most frequently in request from our shelves. Those who once had an ardour for him are not likely ever wholly to lose it; and the great "Essay on Comedy" remains, above all the clamour which the perversity of his manner has aroused, the sole English work on that subject which is worthy of it. We should like to have heard Professor Currie Martin's address, and to see if he shares our pet opinion that Meredith should have written for the stage.

### WOMEN MEDICAL MISSIONARIES.

The London Medical Missionary Association, whose interesting magazine, "Medical Missions at Home and Abroad," has never really reached the general public, has been busy for nearly a generation training men for this branch of mission work. To train women also for it a house has been given to the Association to be used as a hostel for twelve students, who will study at the Women's Medical School connected with the Royal Free Hospital. To furnish the house, and supply what may perhaps be called bursaries for students who cannot afford the fees, a sum of £2,000 is asked for, and the current number of "Mercy and Truth" contains a column of facts concerning the demand for missionaries and nurses in different parts of China. That women are entering or about to enter the medical mission field shows to what a great extent the medical side of the work is needed. For, according to the revered tradition of St. Paul, women are still debarred from mission work as such, though they have often been useful in secretarial and nursing work as the wives of missionaries. There are, it appears, 250 mission hospitals in China, and 400 Western doctors, which gives an average of $1\frac{1}{2}$ foreign doctors to each hospital. There are sixteen medical schools, of which nine are attached to missions, two to the Chinese Government, while five are under private management. The final authority for the whole series of figures is the "Chinese Recorder" for April 1918.

### THE DUTCH COLLEGES OF NURSING.

Nearly all the Dutch unions and associations have men and women members. One of the exceptions is the Dutch male nurses' trade union, which has always been in opposition to the Dutch Women-Nurses' Association, "Nosokomos," first, because the majority of the male nurses did not attain a high degree of culture, and second, because they fight for the care of men-patients through men nurses only, whilst the women nurses would not only care for the women patients, but also for the men. The male nurses' trade union has made an attempt for reconciliation. The success of this attempt was nil, because the women nurses would not give up their independence. The third great Dutch association of nurses, the Dutch Nursing Society, which has men and women members, has but little influence. The nursing affairs in Holland are in confusion.

### PAYING PATIENTS IN POOR LAW INSTITUTIONS.

The suggestion that the Thomas' Inn Institution, which was opened by the City of London Guardian, and controlled by the Metropolitan Asylums Board for pregnant women and married women suffering from venereal disease, should allow its occasionally vacant beds to be used by paying patients has been accepted by the Local Government Board.

### THIS WEEK'S DRUG MARKET.

Business seems to become quieter each week, and there are no signs that buyers are getting tired of postponing their purchases. In the meantime the downward tendencies of prices continues, practically all the changes being in that direction. Cocaine hydrochloride is offered at somewhat lower rates. Caffeine is also slightly lower, Acetyl salicylic acid, salicylic acid, benzoic acid, sodium benzoate, ginger, dill seed, pyrogallic acid, and soft paraffin are all offered at lower rates. Saccharin can hardly be much cheaper; the margin between the amount of the duty and the price at which saccharin is quoted is too small to allow of more than a few shillings reduction. The demand for eucalyptus oil has fallen off and the price has a lower tendency. There is no indication at present of an impending renewal of activity.

